# Draft Genome Sequences of 42 Environmental Vibrio vulnificus Strains Isolated from the Northern Gulf of Mexico

**DOI:** 10.1128/MRA.00200-19

**Published:** 2019-07-11

**Authors:** Megan M. Mullis, I-Shuo Huang, Githzette M. Planas-Costas, Reavelyn Pray, Gregory W. Buck, Arian Nassiri, Courtney Fuentes, Lauren Turner, Gabriel D. Ramirez, Joanna B. Mott, Jeffrey W. Turner

**Affiliations:** a Department of Life Sciences, Texas A&M University—Corpus Christi, Corpus Christi, Texas, USA; b Virginia Division of Consolidated Laboratory Services, Department of General Services, Richmond, Virginia, USA; c College of Natural Sciences and Mathematics, California State University, Sacramento, California, USA; University of Delaware

## Abstract

Vibrio vulnificus is a Gram-negative bacterium and an opportunistic pathogen that can cause septicemia or necrotizing fasciitis. Here, we report the draft genome sequences of 42 environmental V. vulnificus strains collected from the northern Gulf of Mexico. These data will allow for more robust comparisons between clinical and environmental strains.

## ANNOUNCEMENT

Vibrio vulnificus is a Gram-negative marine bacterium with a single polar flagellum (1). This bacterium is capable of producing necrotizing fasciitis if it enters the human body through broken skin or may cause sepsis if ingested, usually through the consumption of raw oysters (2). Previous studies have identified clinical and environmental ecotypes based on sequence polymorphism of the 16S rRNA gene (3) and the virulence-correlated gene (*vcg*) (4). Whole-genome sequencing has confirmed the existence of distinct clinical and environmental ecotypes (5), and the comparative analysis of clinical and environmental genomes has aided in the identification of genes likely required for virulence (6, 7). However, virulence remains poorly understood, and the lack of a large public collection of nonpathogenic genomes has been cited as a limitation (8). To address this data gap, we report the draft genome sequences of 42 environmental strains isolated from the northern Gulf of Mexico (NGOM).

The 42 environmental V. vulnificus strains were isolated previously from seven locations in the Texas segment of the NGOM between August 2006 and July 2007 (9). Isolates were cultured at 37°C overnight on tryptic soy blood agar (Remel, San Diego, CA) and confirmed as V. vulnificus with matrix-assisted laser desorption ionization–time of flight (MALDI-TOF) mass spectrometry (MALDI Biotyper; Bruker, Billerica, MA), as described previously (10). Genomic DNA was extracted with a QIAamp DNA minikit (Qiagen, Hilden, Germany) and quantified with a Qubit double-stranded DNA (dsDNA) broad-range (BR) assay kit (Fisher Scientific, Hampton, NH). Multiplexed paired-end libraries were prepared using a Nextera XT index kit (Illumina, San Diego, CA), per the manufacturer’s instructions, with the modification that libraries were normalized by the estimated genome size prior to pooling. Sequencing was completed using the Illumina MiSeq 500-cycle kit (v2) following standard FastQ-only generation protocols to produce 250-bp paired-end reads. Overlapping paired reads were merged with FLASH version 1.2.11 (option -M 300) (11). Adapter sequences and low-quality bases were trimmed using Trim Galore! version 0.4.0 (options -paired -retain_unpaired) (12). Trimmed reads for the 42 genomes were assembled using SPAdes version 3.9.0 (option -k 127) (13). The 42 genomes were also assembled using MaSuRCA version 3.2.8 (default options) (14) using the insert size distribution estimated with BWA (default options) (15). For this purpose, paired reads were aligned to a draft genome (assembled with SPAdes), and the mean insert size and standard deviation were estimated by parsing the final alignment (in SAM format) with an awk script (16). The draft genome assemblies were filtered by length (500-bp cutoff), and assembly metrics (e.g., total genome size [bp], number of contigs, *N*_50_ value, and G+C content [%]) were calculated by QUAST version 4.1 (default options) (17) to determine which assembler (SPAdes or MaSuRCA) produced a higher-quality genome. For the genomes of two strains, 18057 and 18063, MaSuRCA produced higher-quality assemblies. Draft genomes were annotated using the National Center for Biotechnology Information’s (NCBI’s) Prokaryotic Genome Annotation Pipeline (PGAP) (18).

Table 1 shows the assembly metrics for the 42 V. vulnificus genomes, including the overall genome size (bp), number of contigs, *N*_50_ value, and G+C content (%). The availability of these genomes will augment future comparative genomic analyses focused on the differentiation of clinical and environmental strains. Additionally, the availability of these genomes will advance the understanding of the environmental V. vulnificus reservoir.

**TABLE 1:** Summary of the 42 *Vibrio vulnificus* draft genome assemblies from the northern Gulf of Mexico

Isolate	GenBank accession no.	Genome size (bp)	No. of contigs	*N*_50_ value (bp)	G+C content (%)	Sequence coverage (×)	No. of coding genes	Provenance^*a*^
18022	RCGD00000000	4,845,321	65	270,380	46.75	123.8	4,544	Aransas Bay, 2007
18023	RCGC00000000	4,984,587	83	256,006	46.62	97.1	4,664	Bird Island, 2007
18024	RCGB00000000	4,941,116	111	117,910	46.68	96.34	4,540	Bayside, 2007
18025	RCGA00000000	5,240,241	176	90,860	46.47	82.96	4,999	Bayside, 2007
18026	RCFZ00000000	4,844,420	80	233,461	46.64	75.32	4,564	Bayside, 2007
18027	RCFY00000000	4,843,483	84	195,670	46.70	68.94	4,590	Bayside, 2007
18028	RCFX00000000	4,966,197	79	704,086	46.66	63	4,686	Bayside, 2007
18029	RCFW00000000	4,793,233	95	136,022	46.64	58.78	4,566	Bayside, 2007
18030	RCFV00000000	4,979,258	119	141,772	46.63	40.66	4,653	Bayside, 2007
18031	RCFU00000000	4,850,485	92	130,871	46.70	95.98	4,593	Bayside, 2007
18032	RCFT00000000	4,801,036	113	141,861	46.70	107	4,496	Bayside, 2007
18033	RBZB00000000	5,055,915	162	80,566	46.69	53.08	4,849	Bayside, 2006
18034	RBZD00000000	4,823,242	63	344,960	46.68	102.24	4,471	Bayside, 2006
18035	RBZH00000000	5,240,024	158	94,332	46.49	51.7	4,953	Bayside, 2006
18036	RBZG00000000	4,873,384	71	185,180	46.69	135.3	4,526	Bayside, 2006
18037	RBZF00000000	5,094,022	170	74,007	46.43	119.22	4,720	Copano Bay, 2007
18038	RBZE00000000	5,195,336	252	59,128	46.40	112.54	4,850	Copano Bay, 2007
18039	RBZJ00000000	4,988,278	99	208,583	46.61	58.08	4,678	Copano Bay, 2007
18040	RBZK00000000	4,840,145	79	145,069	46.69	139.02	4,450	Copano Bay, 2007
18041	RBZL00000000	5,412,903	82	235,674	46.51	86.9	5,035	Copano Bay, 2007
18042	RBZC00000000	4,933,479	57	393,935	46.82	104.6	4,492	Copano Bay, 2007
18043	RBZI00000000	4,905,570	90	202,561	46.66	60.38	4,614	Copano Bay, 2007
18044	RPGM00000000	4,935,504	82	207,050	46.63	59.44	4,706	Copano Bay, 2007
18045	RHHD00000000	4,800,809	82	147,435	46.77	42.42	4,469	Copano Bay, 2007
18047	RHHE00000000	4,820,655	103	125,851	46.78	41.88	4,429	Copano Bay, 2007
18048	RHHF00000000	5,082,584	94	209,253	46.72	144.84	4,816	Copano Bay, 2007
18049	RHHG00000000	4,907,792	104	224,385	46.66	99.46	4,612	Copano Bay, 2007
18050	RHHH00000000	4,957,444	99	210,035	46.69	89.38	4,663	Copano Bay, 2007
18051	RHHI00000000	4,927,428	75	187,128	46.79	52.24	4,496	Copano Bay, 2007
18052	RHHJ00000000	5,130,949	88	202,763	46.70	121.64	4,867	Copano Bay, 2007
18053	RPGN00000000	4,884,811	73	204,930	46.88	47.54	4,479	Copano Bay, 2007
18054	RHHK00000000	4,778,453	93	166,931	46.72	68.9	4,489	Copano Bay, 2006
18055	RHHL00000000	4,842,045	79	198,581	46.69	127.62	4,519	Copano Bay, 2006
18056	RBWJ00000000	4,929,131	44	304,520	46.86	105.94	4,490	Copano Bay, 2007
18057	RBWK00000000	4,931,307	79	213,852	46.71	132.62	4,699	Cole Park, 2007
18058	RBWL00000000	4,911,551	97	158,422	46.64	72.88	4,576	Cole Park, 2006
18059	RBWM00000000	4,799,654	65	294,393	46.76	144.34	4,479	Cole Park, 2006
18060	RBWN00000000	4,990,343	46	375,098	46.75	98.92	4,617	Nueces Bay, 2007
18061	RBWO00000000	5,081,056	36	450,849	46.56	119.28	4,721	Nueces Bay, 2007
18062	RBWP00000000	4,923,184	51	390,616	46.83	124.3	4,484	Nueces Bay, 2007
18063	RBWQ00000000	4,929,780	77	188,289	46.71	166.44	4,680	Redfish Bay, 2007
18064	RBWR00000000	5,057,894	117	130,275	46.70	90.8	4,816	Redfish Bay, 2006

a Provenance provided as location and year of collection. All locations are bodies of water in Texas.

### Data availability.

These whole-genome shotgun projects have been deposited at GenBank under the accession numbers listed in Table 1. The raw sequence reads were deposited in the Sequence Read Archive under BioProject accession number PRJNA475262.
